# Defining postnatal growth failure among preterm infants in Indonesia

**DOI:** 10.3389/fnut.2023.1101048

**Published:** 2023-03-13

**Authors:** Rinawati Rohsiswatmo, Risma Kerina Kaban, Muhamad Azharry Rully Sjahrulla, Hardya Gustada Hikmahrachim, Putri Maharani Tristanita Marsubrin, Rosalina Dewi Roeslani, Adhi Teguh Perma Iskandar, Distyayu Sukarja, Ahmad Kautsar, Ivo Urwah

**Affiliations:** Division of Perinatology, Department of Child Health, Faculty of Medicine Universitas Indonesia, Cipto Mangunkusumo General Hospital, Jakarta, Indonesia

**Keywords:** preterm infant, malnutrition, postnatal growth failure, Indonesia, indicator

## Abstract

**Background:**

Postnatal growth failure (PGF) frequently occurred among preterm infants with malnutrition. The decline in a weight-for-age *z*-score of ≥1.2 has been proposed to define PGF. It was unknown whether this indicator would be useful among Indonesian preterm infants.

**Methods:**

Infants of <37 weeks of gestational age born between 2020 and 2021, both stable and unstable, were recruited for a prospective cohort study during hospitalization in the level III neonatal intensive care unit at the Cipto Mangunkusumo General Hospital, Jakarta, Indonesia. The prevalence of PGF as defined by a weight-for-age *z*-score of <−1.28 (<10th percentile) at discharge, a weight-for-age *z*-score of <−1.5 (<7th percentile) at discharge, or a decline in a weight-for-age *z*-score of ≥1.2 from birth till discharge was compared. The association between those PGF indicators with the preterm subcategory and weight gain was assessed. The association between the decline in a weight-for-age *z*-score of ≥1.2 with the duration to achieve full oral feeding and the time spent for total parenteral nutrition was analyzed.

**Results:**

Data were collected from 650 preterm infants who survived and were discharged from the hospital. The weight-for-age *z*-score of <−1.28 or <−1.5 was found in 307 (47.2%) and 270 (41.5%) subjects with PGF, respectively. However, both indicators did not identify any issue of weight gain among subjects with PGF, questioning their reliability in identifying malnourished preterm infants. By contrast, the decline in a weight-for-age *z*-score of ≥1.2 was found in 51 (7.8%) subjects with PGF, in which this indicator revealed that subjects with PGF had an issue of weight gain. Next, a history of invasive ventilation was identified as a risk factor for preterm infants to contract PGF. Finally, the decline in a weight-for-age *z*-score of ≥1.2 confirmed that preterm infants with PGF took a longer time to be fully orally fed and a longer duration for total parenteral nutrition than the ones without PGF.

**Conclusion:**

The decline in a weight-for-age *z*-score of ≥1.2 was useful to identify preterm infants with PGF within our cohort. This could reassure pediatricians in Indonesia to use this new indicator.

## Introduction

Neonatal nutritional care is aimed to maintain postnatal growth linear to normal intrauterine growth (1). Preterm infants with various morbidities are prone to malnutrition, which results in poor growth or even postnatal growth failure (PGF) (2). The risk of postnatal malnutrition is related to decreased nutrient stores at birth, organ immaturity and reduced nutrient absorption, existing comorbidities, dependence on correct identification of malnutrition among infants, and timing of adequate nutrient provision (2). It is, therefore, prudent to correctly identify preterm infants who are malnourished to mitigate or even prevent the potential long-term sequelae of PGF, such as poor neurodevelopment (3).

Various growth parameters have been used to identify infants with PGF, including a weight-for-age *z*-score of <−2.0 at term and 8-month corrected age (4), a weight-for-age *z*-score of <−1.28 (<10th percentile) (5), and a weight-for-age *z*-score of <−1.5 (<7th percentile) at discharge or at approximately 36–40 weeks of postmenstrual age (6). However, those parameters are problematic in accurately identifying preterm infants with growth problems and in predicting their negative adverse outcome (3). Those one-time weight-based indicators have several limitations, including (i) not being predictive of adverse neurodevelopment and (ii) not considering normal postnatal weight loss (3). Other indicators of malnutrition for preterm infants and neonates that focus on a growth pattern (instead of one-time data) had recently been proposed, such as a decline in weight-for-age *z*-score. This indicator is based on an observation that infants with uncomplicated postnatal adaptation or no malnutrition should have a decline in a weight-for-age *z*-score of <0.8 from birth till discharge (2). In addition, this indicator with relatively small cutoffs for mild, moderate, and severe malnutrition suggests that uncomplicated preterm infants are expected to gain weight very rapidly (2). As this indicator is based on differences between the *z*-score at birth and *z*-score at discharge (as opposed to one-time indicators), it has the potential to be more useful in defining PGF among preterm infants. However, as the characteristics of preterm populations might differ between countries, due to genetic variation, social behavior, and existing medical facilities, it is important to assess this new indicator in various preterm populations.

We recently initiated a prospective cohort study in Jakarta, Indonesia [the Cohort of Indonesian PreTerm infants for long-term Outcomes (CIPTO) study] to study preterm infants born at the Cipto Mangunkusumo General Hospital. The CIPTO study is the first and expected to be the largest prospective pediatric cohort in Indonesia. The aims of the CIPTO study were determining the outcomes of those ex-preterm infants and generating an evidence-based reference of preterm care to achieve optimum outcomes. The CIPTO study will be a long-term study as it follows the ex-preterm infants until 8 years old (i.e., school-age children). Hereby, the first part of the CIPTO study was reported, investigating the usefulness of the new indicator of malnutrition (i.e., decline in weight-for-age *z*-score), as compared with common one-time weight-for-age indicators, to identify PGF among Indonesian preterm infants. We observed that this new indicator was useful to define PGF among Indonesians as well.

## Methods

### Study design

The Cohort of Indonesian PreTerm infants for long-term Outcomes (CIPTO) study was a prospective cohort study conducted to observe preterm infants born between 2020 and 2030 at the Cipto Mangunkusumo General Hospital, Jakarta, Indonesia (article of its study protocol is in submission). This report was a part of the CIPTO study, presenting the observational results on preterm infants born between 2020 and 2021 during their perinatal care in the hospital. The Cipto Mangunkusumo General Hospital is a teaching hospital that serves as the national reference hospital and has a level III neonatal intensive care unit (NICU) and greater coverage of diagnosis-related group reimbursement from the National Health Insurance. In addition, this hospital is in Jakarta, the capital city of Indonesia, in which its population comprises various ethnic groups living in the Indonesian archipelago. We recruited preterm infants (<37 weeks of gestational age) who were born during the period of 2020–2021, both stable and unstable, and who survived the neonatal care period and are living in Jakarta and its greater area. The exclusion criteria were parents unwilling to participate, self-discharged before receiving a recommendation from pediatricians, or parents planning to relocate from Jakarta in the subsequent 2 years. The dropout criterion was subjects deceased during the neonatal period. Anthropometric measurement was performed by a trained nurse, nutritionist, or physician. Calibrated tools were used to measure weight. Ethical approval was granted by the Ethical Committee of Faculty of Medicine Universitas Indonesia and Cipto Mangunkusumo General Hospital.

### Exposures and outcomes

The main outcome of this analysis was the incidence of PGF at discharge. We compared the currently used indicators of PGF (i.e., weight-for-age *z*-score below either −1.28 [<10th percentile] or −1.5 [<7th percentile] at discharge without considering the birth weight value) with the new indicator (i.e., a decline in weight-for-age *z*-score ≥ 1.2 from birth to discharge). All indicators are based on the Fenton preterm growth charts. Subgroups of no malnutrition, mild malnutrition, moderate malnutrition, and severe malnutrition were classified based on the decline in weight-for-age *z*-score of <0.8, 0.8–<1.2, 1.2–<2.0, and > 2.0 standard deviation (SD), respectively (2). For this analysis, we used the decline in a weight-for-age *z*-score of ≥1.2 to define PGF. Potential predictors in our analysis were gestational age, birth weight, type of enteral feeding, and comorbidities related to prematurity. Diagnosis of comorbidities was based on current literature and was decided by the attending neonatologists. We measured the association between several growth parameters preceding PGF (weight gain, time to full enteral feeding, and duration of parenteral nutrition) and those weight-based indicators of PGF. Measurement of average weight gain was assessed with methods of early 1-point, average 2-point average, and exponential 2-point (7). The formula to calculate early 1-point was (W_2_−W_1_)/(W_1_/1000)/number of days. The formula to calculate the average 2-point average was (W_2_−W_1_)/[(W_2_ + W_1_)/2]/1,000/number of days. The formula to calculate exponential 2-point was 1,000 × ln (W_2_/W_1_) / number of days, where W_1_ indicates weight at the earliest time point and W_2_ indicates weight at the latest time point. The unit was gram per kilogram per day. Full-enteral feeding was defined as enteral feeding above 120 kcal/kg/day. For infants born <32 weeks, aggressive parenteral nutrition was initiated from birth, but it was not mandatory for infants born >32 weeks.

### Statistical analyses

Data analyses were performed using the STATA software version 15 for Windows. Descriptive statistics was presented according to the type of data. For continuous data, the difference between the two groups was calculated using the Mann–Whitney U-test, while those with more than two groups were calculated using the Kruskal–Wallis test, which was further confirmed by *post hoc* Dunn’s test. Categorical data were analyzed using the chi-square test and Cohen’s kappa test as well as Cox regression for a multivariate analysis. A statistical significance is considered with a *p*-value of <0.05.

## Results

A total of 650 preterm infants were recruited during the study period (Table 1). There were 367 (56.5%) male and 283 (43.5%) female infants who participated in this study. The average gestational age of the subjects at the beginning of the study was 33.1 weeks, while the average gestational age at discharge was 36 weeks. The average birth weight, birth length, and birth head circumference of the subjects were 1,898 g, 41.2 cm, and 29.8 cm, respectively. The majority of subjects received either a mixture of breast milk and formula milk (48.6%) or exclusively breast milk (40.3%). The most and least frequent comorbidities were early-onset sepsis (22.3%) and bronchopulmonary dysplasia (3.7%).

**Table 1:** Demographic data of the study participants.

Characteristics	*n*	%
Sex		
Boy	367	56.5
Girl	283	43.5
Gestational age (week)		
Mean (SD)	33.1 (2.3)	-
Median (min-max)	33 (26–36)	-
Birth weight (gram)		
Mean (SD)	1,898 (497)	-
Median (min-max)	1,855 (720–4,680)	-
Birth length (centimeter)		
Mean (SD)	41.2 (3.8)	-
Median (min-max)	41 (39–54)	-
Birth head circumference (centimeter)		
Mean (SD)	29.8 (2.6)	-
Median (min-max)	30 (28–31)	-
Type of enteral feeding		
Breast milk only	262	40.3
Breast milk and formula	316	48.6
Formula only	72	11.1
Gestational age at discharge (week)		
Mean (SD)	36 (1.8)	-
Median (min-max)	36 (35–43)	-
Length of hospitalization (day)		
Mean (SD)	27 (16)	-
Median (min-max)	22 (9–79)	-
Comorbidities		
Early-onset sepsis	145	22.3
Late-onset sepsis	62	9.5
Necrotizing enterocolitis	38	5.9
History of invasive ventilation	58	8.9
Bronchopulmonary dysplasia	24	3.7
Intraventricular hemorrhage	27	4.2

SD, standard deviation.

The decline in a weight-for-age *z*-score of ≥1.2 SD was chosen with the premise that preterm infants with moderate and severe malnutrition were the ones who were likely to suffer from PGF (2). This indicator was compared with two common weight-for-age indicators of PGF, i.e., a weight-for-age *z*-score of <−1.28 (<10th percentile) and a weight-for-age *z*-score of <−1.5 (<7th percentile), to assess the frequency of PGF within three subcategories of preterm (Table 2). The number of preterm infants with PGF as defined by common weight-based indicators was arguably high (*n* = 307 [47.2%] and *n* = 270 [41.5%] based on a weight-for-age *z*-score of <−1.28 and <−1.5, respectively). This contrasted with the numbers indicated by the decline in a weight-for-age *z*-score of ≥1.2 (*n* = 51 [7.8%]). This difference was presumably contributed by a strong tendency of those common weight-based indicators to define PGF among moderate-to-late preterm infants (81.4 and 80.4%) but a not too strong tendency based on the decline in a weight-for-age *z*-score of ≥1.2 (52.9%).

**Table 2:** Incidence of postnatal growth failure across subcategories of preterm.

Subcategory of Preterm	Indicator of Postnatal Growth Failure					
	Weight *z*-score < −1.28		Weight *z*-score < −1.5		Decline in weight-for-age *z*-score ≥ 1.2	
	*n*	%	*n*	%	*n*	%
Moderate-late preterm (*n* = 518)	250	48.3	217	41.9	27	5.2
Very preterm (*n* = 113)	49	43.4	45	39.8	18	15.9
Extreme preterm (*n* = 19)	8	42.1	8	42.1	6	31.6

Chi-square result for the column of weight *z*-score < −1.28 was *p* = 0.577. Chi-square result for the column of weight *z*-score < −1.5 was *p* = 0.920. Chi-square result for the column of decline in a weight-for-age *z*-score of ≥1.2 was *p* = 0.000. Cohen’s kappa statistic: Weight *z*-score < −1.28 versus decline in a weight-for-age *z*-score of ≥1.2: kappa 0.071; agreement 61.09%. Weight *z*-score < −1.5 versus decline in a weight-for-age *z*-score of ≥1.2: kappa 0.085; agreement 65.95%. Percentages were calculated per subcategory of preterm. Weight *z*-score < −1.28 is weight < 10th percentile. Weight *z*-score < −1.5 is weight < 7th percentile.

Preterm infants with PGF by definition should accumulate less weight (2). In contrast to a weight-for-age *z*-score of <−1.28 and <−1.5, the decline in a weight-for-age *z*-score of ≥1.2 could better distinguish preterm infants with PGF from the ones with no PGF in terms of weight gain (Table 3). Irrespective of the calculation methods, preterm infants with PGF identified by a decline in a weight-for-age *z*-score of ≥1.2 accumulated less weight as compared with the ones without PGF (*p* < 0.05). Intriguingly, a paradoxical association was observed between a weight-for-age *z*-score of <−1.28 and <−1.5 with weight gain, in which PGF preterm infants identified by these indicators gained more weight than the non-PGF ones (*p* < 0.001). These findings, indeed, question the reliability of those current one-time weight-based indicators to define preterm infants with PGF (2).

**Table 3:** Association between various weight-based indicators of postnatal growth failure and weight gain during admission in neonatal care.

Indicator	Early 1-point (g/kg/day)			Average 2-point average (g/kg/day)			Exponential 2-point (g/kg/day)		
	PGF	No PGF	*p* value	PGF	No PGF	*p* value	PGF	No PGF	*p* value
Weight *z*-score < −1.28	8.79 (4.14–14.38)	5.72 (0.00–13.61)	<0.001	8.04 (4.03–11.35)	5.43 (0.00–11.06)	<0.001	8.07 (4.03–11.46)	5.14 (0.00–11.12)	<0.001
Weight *z*-score < −1.5	8.89 (4.55–14.77)	5.74 (0.00–13.60)	<0.001	8.05 (4.37–11.53)	5.48 (0.00–11.05)	<0.001	8.08 (4.37–11.84)	5.20 (0.00–11.11)	<0.001
Decline in weight-for-age *z*-score ≥ 1.2	4.44 (−1.04–9.40)	8.04 (0.00–14.11)	<0.001	4.22 (−1.05–8.04)	7.43 (0.00–11.39)	<0.001	4.44 (−1.04–9.40)	8.04 (0.00–14.11)	<0.001

Data were presented as median (25th–75th percentile). Measurement of average weight gain was assessed by using three formulas. Formula to calculate early 1-point = (W2−W1)/(W1/1000)/number of days. Formula to calculate average 2-point average = (W2−W1)/[(W2 + W1)/2]/1,000/number of days. Formula to calculate exponential 2-point = 1,000 × ln (W2/W1) / number of days. W1 indicates weight at the earliest time point. W2 indicates weight at the latest time point. The Mann–Whitney U-test was used to compare the medians from PGF and no PGF in the same row. Weight *z*-score < −1.28 is weight of <10th percentile. Weight *z*-score < −1.5 is weight of <7th percentile. PGF, postnatal growth failure.

Next, analyses of several potential risk factors to develop PGF, as defined by those weight-based indicators, were conducted (Table 4). First, as compared with preterm infants who exclusively received breast milk, no statistical difference in terms of risk to develop PGF was observed among preterm infants who were fed with formula milk only. Intriguingly, the decline in a weight-for-age *z*-score of ≥1.2 (RR = 0.28; 95% CI:0.12–0.65; *p* = 0.003), but not other indicators, suggested that the preterm infants who received a combination of breast milk and formula milk had a lower risk than the ones who exclusively received breast milk to develop PGF. Second, several comorbidities (i.e., early-onset neonatal sepsis; late-onset neonatal sepsis; necrotizing enterocolitis; bronchopulmonary dysplasia; and intraventricular hemorrhage) were prominent in not increasing the risk of PGF, irrespective of the definitions. However, while a weight-for-age *z*-score of <−1.28 and <−1.5 did not identify any statistical difference in the history of invasive ventilation, the decline in a weight-for-age *z*-score of ≥1.2 suggested that preterm infants with a history of invasive ventilation had a higher risk to develop PGF (RR = 3.04; 95% CI:1.41–6.53; *p* = 0.004). Collectively, the decline in a weight-for-age *z*-score of ≥1.2 was able to define the population with PGF as well as its associated risk factor.

**Table 4:** Multivariate analysis on risk factors associated with postnatal growth failure.

Risk factor	Weight *z*-score < −1.28		Weight *z*-score < −1.5		Decline in weight-for-age *z*-score ≥ 1.2	
	RR	95% CI	RR	95% CI	RR	95% CI
Enteral feeding (versus breast milk)						
Breast milk and formula	0.84	0.64–1.09	0.79	0.60–1.05	**0.28**	**0.12–0.65**
Formula only	0.86	0.58–1.27	0.80	0.52–1.22	0.90	0.37–2.19
Early-onset neonatal sepsis	0.93	0.66–1.32	0.91	0.63–1.33	2.11	0.93–4.79
Late-onset neonatal sepsis	1.01	0.63–1.63	0.89	0.52–1.50	1.97	0.76–5.13
Necrotizing enterocolitis	1.49	0.94–2.38	1.55	0.95–2.53	0.54	0.19–1.54
Bronchopulmonary dysplasia	0.97	0.52–1.79	1.05	0.57–1.96	1.98	0.83–4.73
Intraventricular hemorrhage	0.58	0.28–1.18	0.58	0.27–1.23	0.94	0.29–3.11
History of invasive ventilation	1.04	0.65–1.64	1.13	0.70–1.83	**3.04**	**1.41–6.53**

Weight *z*-score < −1.28 is weight of <10th percentile. Weight *z*-score < −1.5 is weight of <7th percentile. RR, relative risk; CI, confidence interval. Bold letters indicate a value of *p*-value of <0.05.

**Figure 1:**
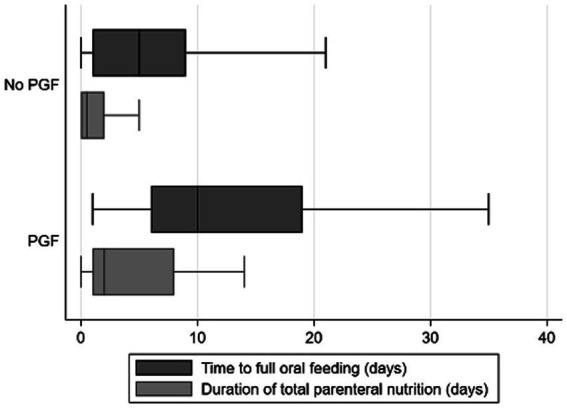
Association between decline in a weight-for-age *z*-score of ≥1.2 and time to achieve full oral feeding as well as time spent for total parenteral nutrition. Box and whisker plots were presented for time to full oral feeding (in days; represented by dark gray bars) and time duration of total parenteral nutrition (in days; represented by light gray bars) and as defined by the decline in weight-for-age *z*-scores of <1.2 (“No PGF”) and ≥1.2 (“PGF”). The box contains the 25th to 75th percentile of the dataset, while the central line within the boxes denotes the median value. The whiskers mark the minimum and maximum datasets.

The association between the decline in weight-for-age *z*-score and duration of total parenteral nutrition or time to achieve full oral feeding was finally analyzed. Preterm infants identified by the decline in a weight-for-age *z*-score of ≥1.2 had a longer duration of total parenteral nutrition and the time to achieve full oral feeding, as compared with the ones identified by the decline in *z*-scores of <1.2 (Figure 1; median of total parenteral nutrition = 2 vs. 0.5 days; median of achieving full oral feeding = 10 vs. 5 days). Taken together, this finding supports the usefulness of the decline in a weight-for-age *z*-score of ≥1.2 to identify preterm infants with PGF.

## Discussion

We hereby report the first part of the CIPTO study, focusing on 650 infants of <37 weeks of gestational age at birth between 2020 and 2021 at the perinatal unit of Cipto Mangunkusumo General Hospital, Jakarta, Indonesia. Our results could be summarized into three points. First, the decline in a weight-for-age *z*-score of ≥1.2 (indicating moderate and severe malnutrition) was useful to identify preterm infants with PGF in this cohort. While the decline in a weight-for-age *z*-score of ≥1.2 identified only 51 subjects with PGF, a weight-for-age *z*-score of <−1.28 and <−1.5 somehow identified 250 and 217 subjects with PGF, respectively. We argue that this was an issue of overestimation, as a weight-for-age *z*-score of <−1.28 and <−1.5 reported that subjects with PGF in our cohort did not have any issue with weight accumulation, as compared with the subjects without PGF. This interpretation was in line with a recent challenge to the usefulness of a weight-for-age *z*-score of <−1.28 as an indicator of PGF (3) due to its inability to predict any adverse outcome and its inability to adequately identify malnourished preterm infants. The latter notion could result in nutrition intake above infants’ actual requirement, predisposing those infants to later develop metabolic diseases (8). Another supporting finding of the usefulness of the decline in a weight-for-age *z*-score of ≥1.2 to identify preterm infants with PGF was that the identified subjects had a protracted time receiving parenteral nutrition or a delayed time in receiving full enteral nutrition.

Second, we observed that formula feeding or breast milk plus formula feeding did not increase the risk of PGF as compared with exclusive breast milk feeding. This finding suggests that, irrespective of the mode of enteral feeding, timely initiation of enteral feeding with adequate calories was the important factor to prevent malnutrition and PGF among preterm infants (9, 10). An interesting observation from our cohort was that breast milk plus formula feeding was associated with a lower risk to develop PGF than exclusive breast milk feeding when evaluated by the decline in a weight-for-age *z*-score of ≥1.2. Exclusive breast milk feeding for 6 months is a well-known fact to support infants to be healthy and to grow and develop adequately. However, if exclusive breast milk feeding is not possible or is insufficient for optimal growth and development, we believe that the mode of breast milk plus formula feeding or the nutrient fortification of breast milk would allow preterm infants to enjoy the immuno-nutrient advantage of breast milk as well as the higher amounts of important nutrients of formula (11, 12). This could help to reduce the risk to develop PGF. It is important to consider, however, that this interpretation might be skewed due to the low numbers of infants receiving exclusive breast milk feeding at the NICU. It is commonly acknowledged that rates of breastfeeding initiation and duration were lower among infants treated at the NICU, particularly in developing countries, due to various reasons, including unstable infant condition in the first 24–48 h of life, maternal health issues, and anguish related to their hospitalized infants, the detachment between mothers and infants due to physical separation, lack of maternal privacy to lactate, and inadequate support for exclusive breast milk feeding (13–18).

Finally, we noted that the decline in a weight-for-age *z*-score of ≥1.2 identified that a history of invasive ventilation was associated with a higher risk among preterm infants to develop PGF. This is not surprising since the history of invasive ventilation, in particular, the prolonged one, increased the risk of respiratory morbidity (e.g., bronchopulmonary dysplasia) (19, 20), which could result in a higher risk of PGF (19–21).

Postnatal growth failure is commonly caused by pediatric malnutrition, in which preterm infants are prone to suffer from nutritional deficits (2). This implies the importance to have a standardized indicator to identify malnutrition among preterm infants as a surrogate indicator of PGF, which can be properly utilized by pediatricians worldwide (3). Various data could be used to identify malnutrition among preterm infants, comprising changes in weight, length, head circumference, body composition, body mass index, mid-upper arm circumference, and nutrition-focused physical exam (2). Weight and its changes are the most common indicator to be used as preterm infants with adequate nutrition should gain weight rapidly and as these data could be accurately collected (2). It should be noted, however, that weight measurement alone might not accurately identify infants who had weight gain disproportionate to their growths in length and head circumference (16, 17). Hence, some experts recommended calculating the decline in both length-for-age and head circumference-for-age *z*-scores as well, to complement the indicator of decline in weight-for-age *z*-score (2). Nonetheless, as length and head circumference assessments are only useful if they are accurately measured, the same experts proposed that the decline in weight-for-age *z*-score could be used as a single primary indicator to diagnose PGF (2).

A good definition of PGF allows early detection of the true population-at-risk, which thus could minimize problems with overdiagnosis and intervention-related adverse risk, such as risks for obesity and cardiovascular disease in later life due to overfeeding (22). It was concerning, therefore, to observe a trend of PGF overdiagnosis within our cohort when the single-point definitions of a weight-for-age *z*-score of <−1.28 and <−1.5 at discharge had been deployed. A recent review by Fenton et al (3), indeed, challenged the usefulness of a weight-for-age *z*-score of <−1.28 as the obtained result was solely based on the weight, was measured without correction, and was not predictive of poor neurodevelopment. A similar argument was presented by Zozaya et al (6) when they observed that a weight-for-age *z*-score of <−1.5 at 36 weeks was not associated with a worse Bayley II mental development index. By contrast, the definitions of decline in a weight-for-age *z*-score of ≥1.2 (2) provided a useful estimation of PGF within our cohort, corroborated by association with issues of weight accumulation and a history of invasive ventilation. It is important to note, however, that the accuracy of the definition is influenced by the growth charts as well. The INTERGROWTH-21st Preterm Postnatal Growth Standards curves were created as a better alternative than size-at-birth charts (23, 24), in which a recent study suggested an alternative definition of PGF (i.e., a decline in a weight-for-age *z*-score of 1.0 or higher) based on INTERGROWTH-21st growth curves to predict the risk of cognitive delay (25). It would be of interest in future to assess PGF among preterm infants by using this alternative growth curve.

There were several limitations found in our study. One limitation is that it was a single-center study design with a small number of infants born at 26–27 weeks (i.e., extremely preterm) recruited in this report. Our cohort did not have preterm infants born below 26 weeks. Thus, the reliability of our data among extremely preterm infants might be questionable. In addition, we did not stratify our preterm cohort based on any existing comorbidity during the admission at the NICU (i.e., stable vs. unstable preterm infants). Thus, we could not compare the growth trajectory between both groups. Next, as this report described the first part of the ongoing CIPTO study, the complete data on health, growth, and development outcomes of those surviving preterm infants at 24 months and beyond are unavailable yet. This limits our ability to calculate sensitivity and specificity, as more concrete outcomes, in evaluating those weight-based indicators of PGF among preterm infants. Another limitation is that our current cohort does not have comparable proportions of categories such as small for gestational age, appropriate for gestational age, and large for gestational age. Thus, we could not draw any conclusion regarding the impact of weight-for-gestational age at birth on the incidence and outcomes of preterm infants with PGF. We also acknowledge the possibility that different timings of discharge from the NICU might contribute to potential differences observed within our cohort since we did not stratify our cohort based on the timing of hospital discharge. Nonetheless, as the hospital discharge was primarily based on clinical judgment (i.e., age ≥ 36 weeks postmenstrual age, weight > 2,000 g, and no more indication for intensive monitoring), we hypothesize that the impact of timing differences in hospital discharge would be minimum. Finally, this part of the CIPTO study only assessed weight-based indicators to define PGF. It is known that chronic inadequate nutrient intake would also reduce length gain and head growth. This suggests that weight-based indicators should be accompanied by length- and head circumference-based indicators to define PGF well among preterm infants.

Further studies should be conducted based on the CIPTO cohort, such as (i) assessing the health, growth, and developmental outcomes of those surviving preterm infants, with and without PGF, at 24 months and beyond; (ii) comparing the impact of weight-for-gestational age at birth on the incidence and outcomes of preterm infants with PGF; (iii) complementing the weight-based indicator with length- and head circumference-based indicators to define PGF among preterm infants; and (iv) determining the preterm growth curve (e.g., the Fenton vs. INTERGROWTH-21st charts) that suits best in defining PGF among preterm infants.

In conclusion, the decline in a weight-for-age *z*-score of ≥1.2 was useful to define preterm infants with PGF within our cohort. This could provide reassurance to pediatricians in Indonesia for switching from the common-yet-problematic indicators, such as a weight-for-age *z*-score of <−1.28 or <−1.5, to this new indicator to identify preterm infants with PGF.

## Data availability statement

The original contributions presented in the study are included in the article/supplementary material, further inquiries can be directed to the corresponding author.

## Ethics statement

The studies involving human participants were reviewed and approved by the ethics committee, Faculty of Medicine, Universitas Indonesia. Written informed consent to participate in this study was provided by the participants’ legal guardian/next of kin.

## Author contributions

The primary investigators were RR and HH. The primary data coordinator was IU. The main coordinator for data assessment and collection during the in-hospital period was MS. All other co-authors were involved in conducting this study. All authors contributed to the article and approved the submitted version.

## Funding

All funding related to inpatient care are provided by national health insurance. Routine health visits in the community setting are a part of the Indonesian Ministry of Health’s program, thus fully funded by the national health insurance. However, the cost of visiting a pediatrician at a private clinic or a hospital would be borne by subjects individually. This cohort was partially funded by PT Sarihusada Generasi Mahardika. It had no role in study design and data analyses, and it did not support any product related to preterm infant care during hospital admission or after hospital discharge. It did not receive any access to the research data and participants’ personal information.

## Conflict of interest

The authors declare that the study was conducted in the absence of any commercial or financial relationships that could be construed as a potential conflict of interest.

## Publisher’s note

All claims expressed in this article are solely those of the authors and do not necessarily represent those of their affiliated organizations, or those of the publisher, the editors and the reviewers. Any product that may be evaluated in this article, or claim that may be made by its manufacturer, is not guaranteed or endorsed by the publisher.
